# The Spike Glycoprotein of SARS-CoV-2 Binds to β1 Integrins Expressed on the Surface of Lung Epithelial Cells

**DOI:** 10.3390/v13040645

**Published:** 2021-04-09

**Authors:** Eun Jeong Park, Phyoe Kyawe Myint, Michael Gyasi Appiah, Samuel Darkwah, Siqingaowa Caidengbate, Atsushi Ito, Eri Matsuo, Eiji Kawamoto, Arong Gaowa, Motomu Shimaoka

**Affiliations:** 1 Department of Molecular Pathobiology and Cell Adhesion Biology, Mie University Graduate School of Medicine, 2-174 Edobashi, Tsu-City 514-8507, Mie, Japan; 317DS09@m.mie-u.ac.jp (P.K.M.); 317DS06@m.mie-u.ac.jp (M.G.A.); kwekuadarkwah@gmail.com (S.D.); 320D011@m.mie-u.ac.jp (S.C.); i-atsushi@clin.medic.mie-u.ac.jp (A.I.); ematsuo@med.mie-u.ac.jp (E.M.); a-2kawamoto@med.mie-u.ac.jp (E.K.); arong-g@doc.medic.mie-u.ac.jp (A.G.); 2 Department of Cardiothoracic Surgery, Mie University Graduate School of Medicine, 2-174 Edobashi, Tsu-City 514-8507, Mie, Japan; 3 Department of Emergency and Disaster Medicine, Mie University Graduate School of Medicine, 2-174 Edobashi, Tsu-City 514-8507, Mie, Japan

**Keywords:** SARS-CoV-2, spike 1 protein, angiotensin-converting enzyme 2, integrin, cell adhesion, alveolar epithelial cells

## Abstract

The spike glycoprotein attached to the envelope of severe acute respiratory syndrome coronavirus 2 (SARS-CoV-2) binds to and exploits angiotensin-converting enzyme 2 (ACE2) as an entry receptor to infect pulmonary epithelial cells. A subset of integrins that recognize the arginyl–glycyl–aspartic acid (RGD) sequence in the cognate ligands has been predicted in silico to bind the spike glycoprotein and, thereby, to be exploited for viral infection. Here, we show experimental evidence that the β1 integrins predominantly expressed on human pulmonary epithelial cell lines and primary mouse alveolar epithelial cells bind to this spike protein. The cellular β1 integrins support adhesive interactions with the spike protein independently of ACE2, suggesting the possibility that the β1 integrins may function as an alternative receptor for SARS-CoV-2, which could be targeted for the prevention of viral infections.

## 1. Introduction

Severe acute respiratory syndrome coronavirus 2 (SARS-CoV-2) causes a potentially fatal acute pulmonary infection, leading to a cytokine storm that culminates in acute respiratory distress syndrome and other COVID-19 pathologies [1,2]. As of April 2021, the SARS-CoV-2 pandemic is reported to globally comprise more than 133 million infected cases, including more than 2.8 million deaths [3]. SARS-CoV-2 is a single-strand positive-sense RNA virus that on the surface displays a large spike glycoprotein (S-protein), which plays a critical role in the interaction between the virus and host cells at entry sites. Angiotensin-converting enzyme 2 (ACE2), an ecto-enzyme present on the cell surface, has been identified as a major cellular receptor for the viral spike glycoprotein, one that aids viral entry to host alveolar epithelial cells and other epithelial and endothelial cells [4]. ACE2 is regarded as a promising therapeutic target for blocking such viral entry [4,5]. However, multiple cell-surface receptors other than ACE2 may be simultaneously involved in the process of viral entry [6,7,8,9].

Integrins represent the largest family of cell-adhesion receptors. Composed of the α-and β-subunits that bind to ligands on the opposing cells and in the matrix proteins, these integrins mediate adhesive interactions across a wide range of biological processes including organ development, wound healing and angiogenesis, host defense and inflammation, and hemostasis [10]. In both humans and mice, 18 α-subunits and eight β-subunits have now been identified; both participate in the formation of 24 non-covalently associated α/β heterodimer cell-surface receptors [10]. Several integrins, including the fibronectin receptor α5β1 integrin and the vitronectin receptor αVβ3 integrin, specifically recognize a surface-exposed arginyl–glycyl–aspartic acid (RGD) sequence in the cognate ligands in a Mg^2+^ ion-dependent manner [11]. Recent investigations have revealed that the S-protein of SARS-CoV-2, but not other coronaviruses such as SARS-CoV and MERS-CoV, contains a surface-exposed RGD sequence [12,13,14]. This component can thereby predict the binding of S-protein to the cellular integrins, which would facilitate the viral entry of SARS-CoV-2. Targeting the integrin-binding RGD sequence in the S-protein could be used for blocking SARS-CoV-2 infections [6,15]. Here, we set forth experimental evidence showing that integrins in human pulmonary epithelial cell lines and mouse primary alveolar epithelial cells bind to the spike protein.

## 2. Materials and Methods

### 2.1. Culturing of Different Cell Lines

The MLO-A5 cell line (mouse osteocytes) was obtained from Kerafast (Boston, MA, USA) and grown in MEM alpha (Thermo Fisher Scientific, Waltham, MA, USA) containing 5% fetal bovine serum (FBS; Equitech-Bio, Kerrville, TX, USA), 5% calf serum (Biowest, Riverside, MO, USA), and penicillin/streptomycin (Nacalai, Kyoto, Japan). A human monocytic cell line (THP-1) was purchased from ATCC (Manassas, VA, USA) and human lung epithelial cell lines (11-18 and QG-56) were kindly provided by Dr. Yoshihiro Miyahara (Mie University Medical School, Mie, Japan). The human breast cancer line MDA-MB-231 was from ATCC. β1-control (Scr.) and β1-KO clones of MDA-MB-231 generated in our laboratory [16] were used to validate the effect of β1 integrin on cell adhesion to S1 protein. Cells were grown in RPMI-1640 (Nacalai) containing 10% FBS (Equitech-Bio) and penicillin/streptomycin (Nacalai).

### 2.2. Mice

C57BL/6J mice (10 to 13 weeks old) were obtained from CLEA Japan (Tokyo, Japan). The mice were maintained in the Mie University Experimental Animal Facility under specific pathogen-free conditions with ad libitum access to water and food and with a standard 12-hour light–dark cycle. The experimental animal protocol was approved by the Ethics Review Committee of Mie University (Approval number: #2019-41).

### 2.3. Isolation of Primary Lung Epithelial Cells from Mouse Lungs

Epithelial cells were isolated from mouse lung tissues by using a mechanical dissociation with sterile sieve meshes followed by a discontinuous density-gradient centrifugation using Percoll (GE Healthcare Life Sciences, Chicago, IL, USA) in accordance with previous studies [17,18] with some modifications. Three Percoll solutions (25%, 40%, and 75%) were prepared, the cells from lung tissues were resuspended in 40% solution, and three distinct gradients were carefully placed in the order of 25%–40%–75% from top to bottom in a 15 mL centrifuge tube (Corning, Glendale, AZ, USA). The cells were spun at 780× *g* for 20 min in a centrifuge (AX-511) (Tomy, Tokyo, Japan) at the setting of minimal acceleration and deceleration. The epithelial cells were centered at an interface between 25% and 40% Percoll solution and were then taken and washed extensively with RPMI1640 (Nacalai) containing 10% FBS (Equitech-Bio) and penicillin/streptomycin (Nacalai).

### 2.4. Cell Adhesion Assay

An adhesion assay was carried out as previously described [19]. Ninety-six-well V-bottom plates (Greiner, Tokyo, Japan) were coated with 2.5 μg/mL SARS-CoV-2 spike protein 1, Fc Tag (S1-Fc) (Sino Biological, Wayne, PA, USA), 2.5 μg/mL SARS-CoV-2 spike protein 1, Fc Tag (S1-Fc) (Sino Biological), or 2.5 μg/mL IgG Fc fragment protein (Abcam, Cambridge, MA, USA) at 4 °C for 18 h and washed twice with phosphate-buffered saline (PBS). To chelate the metal ions, cells were resuspended in serum-free medium containing 5 mM ethylenediaminetetraacetic acid (EDTA) (Wako, Osaka, Japan) and washed twice with the same medium with no EDTA. The cells were then labeled with a fluorescent dye (1 mM 3′-O-Acetyl-2′,7′-bis(carboxyethyl)-4 or 5-carboxyfluorescein, diacetoxymethyl ester (BCECF-AM)) (Dojindo, Kumamoto, Japan) and washed with 4-(2-hydroxyethyl)-1-piperazineethanesulfonic acid (HEPES)-buffered saline. Equal numbers of labeled cells (1 to 5 × 10^4^ per well) were plated into the V-bottom wells and incubated with either anti-integrin monoclonal antibodies (mAbs) or isotype controls (1 μg/mL for anti-human integrin and 10 μg/mL for anti-mouse integrin antibodies) at room temperature for 10 min. Anti-mouse CD29 (HMb1-1) mAb and its isotype control (HTK888) for treating mouse cells were obtained from BioLegend (San Diego, CA, USA) and used at a concentration of 10 μg/mL; anti-human CD29 (P5D2) (R&D Systems, Minneapolis, MN, USA) and its isotype (MOPC-21) (Biolegend), anti-mouse/human CD49d (PS/2) (Southern Biotech, Birmingham, AL, USA) and its isotype (G013B8) (Biolegend), and anti-human CD51 (NKI-M9) (Biolegend) and its isotype (MG2a-53) (Biolegend) mAbs were used at 1 μg/mL. The cells were incubated with either 2 mM EDTA (Wako, Osaka, Japan) or 1 mM CaCl_2_ (Sigma-Aldrich) plus 1 mM MgCl_2_ (Sigma-Aldrich) for 10 min. The plates were spun at 280× *g* for 5 min in the centrifuge (AX-511; Tomy Seiko Co. Ltd., Tokyo, Japan). The fluorescence obtained from unbound pellets was measured with a 2030 ARVO reader (PerkinElmer, Waltham, MA, USA). Cell adhesion to S1-Fc and other control proteins such as S2-Fc and IgG Fc fragment is expressed as the percentages of bound cells to input cells, after the application of the centrifugal force to produce shear stress to separate free cells from bound cells [19].

### 2.5. Flow Cytometry

Monoclonal antibodies (mAbs) to human integrins were purchased as follows: β1 (TS2/16) (Biogems, Westlake Village, CA), β3 (VIPL2) (Abcam), β5 (AST-3T) (Biolegend), α5 (NKI-SAM-1) (Biolegend), and αV (P2W7) (LSBio, Seattle, WA, USA). Anti-mouse integrin MAbs were also obtained: β1 (HMb1-1) (Biolegend), β3 (HMb3-1) (Biolegend), β5 (KN52; Thermo Fisher Scientific), α5 (5H10-27) (Biolegend), and αV (RMV-7) (Biolegend). Isotype-matched control antibodies were also obtained: mouse IgG1 (MOPC-21, Biolegend), mouse IgG2a (MOPC-173) (Biolegend), mouse IgG2b (MPC-11) (Biolegend), Armenian hamster IgG (HTK888) (Biolegend), and rat IgG1 (RTK2071) (Biolegend). The cells were stained with those fluorescently labeled antibodies, washed twice with PBS containing 2% FBS and 2 mM EDTA (Wako, Osaka, Japan), and analyzed by using a BD Accuri C6 flow cytometer and software (BD Biosciences, San Jose, CA, USA).

### 2.6. Reverse Transcription and Quantitative Polymerase Chain Reaction (RT-qPCR)

Total RNA was extracted from the cells by using TRIzol reagent (Thermo Fisher Scientific) and RT was conducted with a Prime Script RT Kit (Takara Bio, Shiga, Japan) according to the manufacturer’s instructions. To determine relative *ACE2* expression, qPCR was performed by using a PowerUp SYBR Green Master Mix PCR kit (Applied Biosystems, Foster City, CA, USA) and the StepOne Real-Time PCR System (Applied Biosystems) according to the manufacturer’s instructions. For endogenous controls, *β-actin* was used to normalize *ACE2* expression. The PCR primers (5′→3′) used in this study were as follows: human *β-actin*, CCCTGGACTTCGAGCAAGAG (forward) and ACTCCATGCCCAGGAAGGAA (reverse); human *ACE2*, AAACATACTGTGACCCCGCAT (forward) and CCAAGCCTCAGCATATTGAACA (reverse); mouse *β-actin*, GATCAAGATCATTGCTCCTCCTGA (forward) and AAGGGTGTAAAACGCAGCTCA (reverse); and mouse *ACE2*, TGGGCAAACTCTATGCTG (forward) and TTCATTGGCTCCGTTTCTTA (reverse). Relative expressions were calculated by using the comparative threshold method (2^−ΔCT^) normalized to *β-actin*.

### 2.7. Statistical Analysis

Data are expressed as the mean ± standard error of the mean (SEM) and were analyzed using a two-tailed unpaired *t*-test for comparisons between two groups and a one-way analysis of variance (ANOVA) for comparisons among three groups. *p* values less than 0.05 were considered significant. Statistical analysis was done by using the Prism8 software (GraphPad, San Diego, CA, USA).

## 3. Results

**Figure 1:**
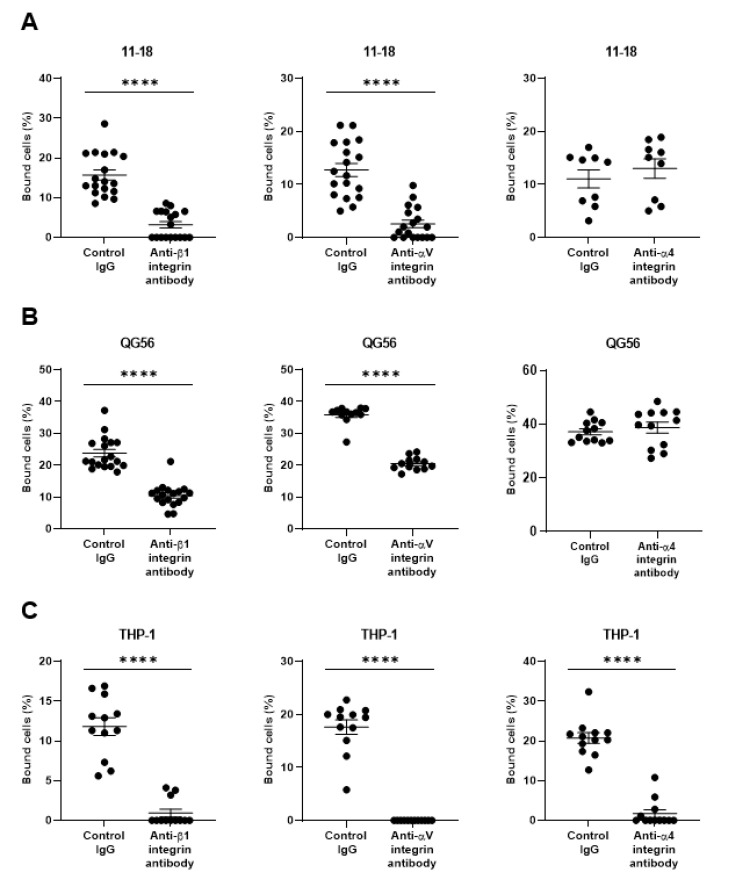
Binding of the SARS-CoV-2 spike (S1) protein to human pulmonary cell lines 11–18 (**A**) and QG-56 (**B**) and to the monocyte-like cell line THP-1 (**C**). Results are shown as column scatterplots overlayed with the mean ± SEM of at least 3 independent experiments, in which each sample was triplicated. **** *p* < 0.0001.

**Figure 2:**
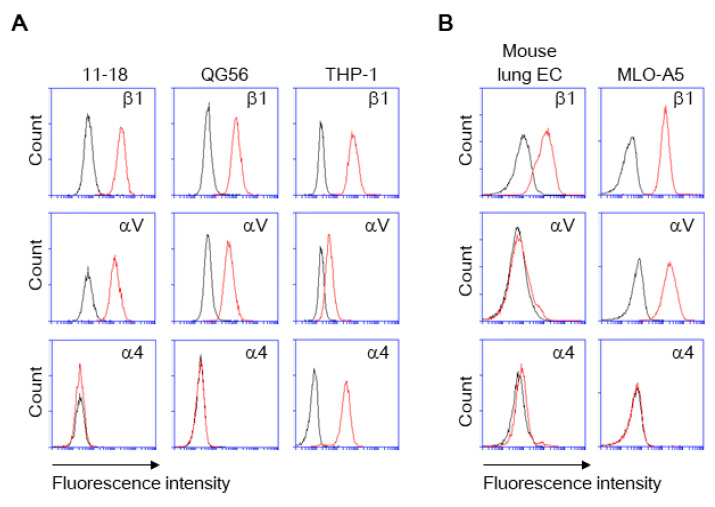
Expression of β1, αV, and α4 integrins on cells. The cells were stained with the fluorescently labeled monoclonal antibodies (mAbs) to integrins. The integrin expression was analyzed by using flow cytometry for human (**A**) and mouse (**B**) cells. Histograms indicate the expression of integrins indicated. Data are representative of three separate experiments. Red lines, mAb; black lines, isotype IgG.

As pulmonary epithelial cells represent a prime target for SARS-CoV-2, we have investigated the binding of the spike protein to the human pulmonary epithelial cell lines 11-18 and QG-56. We have found that these epithelial cell lines showed good binding to the spike protein (Figure 1A,B). β1 integrins are the predominant integrins expressed in pulmonary epithelial cells under physiologic conditions, although some levels of αVβ3, α6β4, αVβ5, αVβ6, and αVβ8 integrins are also expressed (Figure 2) and upregulated under pathologic conditions such as in various cancers and fibrosis [20]. Thus, we have tested the effects of anti-β1 and anti-αV integrin inhibitory antibodies that block all β1 integrins (α1β1, α2β1, α3β1, α4β1, α5β1, α6β1, α7β1, α8β1, α9β1, α10β1, and α11β1) and αV integrins (αVβ1, αVβ3, αVβ5, αVβ6, and αVβ8), respectively. The adhesive interactions of pulmonary epithelial cells with the spike 1 (S1) protein are potently inhibited by pretreatment with anti-β1 or anti-αV integrin inhibitory antibodies (Figure 1A,B). 

As monocytes/macrophages are implicated in the pathogenesis of COVID-19, we have studied human monocyte-like cell line THP-1, thereby showing a good binding to S1 protein (Figure 1C). THP-1 binding to S1 protein was blocked by not only anti-β1 and anti-αV integrin inhibitory antibodies, but the anti-α4 integrin inhibitory antibody that blocks α4β1, and α4β7 integrins (Figure 1C). THP-1 cells express α4 integrin, whereas 11-18 and QG-56 cells do not (Figure 2). Thus, the anti-α4 integrin inhibitory antibody did not show any blocking effects on 11-18 and QG-56 cells (Figure 1A,B).

**Figure 3:**
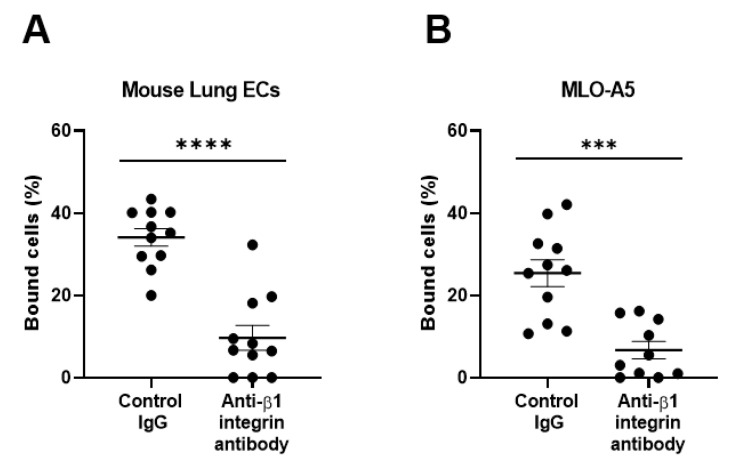
Binding of the SARS-CoV-2 spike (S1) protein to mouse primary lung cells (**A**) and osteoclastic MLO-A5 cells (**B**). Results are shown as column scatterplots overlayed with the mean ± SEM of at least 3 independent experiments, in which each sample was triplicated. *** *p* < 0.001; and **** *p* < 0.0001.

To further substantiate our findings regarding the β1 integrin-dependent binding of spike proteins to pulmonary epithelial cell lines, we studied primary pulmonary cells freshly isolated from mouse lungs that were enriched with alveolar epithelial cells. We have shown that mouse primary lung cells robustly bind to S1 proteins, which are inhibited by the antibody to mouse β1 integrins (Figure 3A).

**Figure 4:**
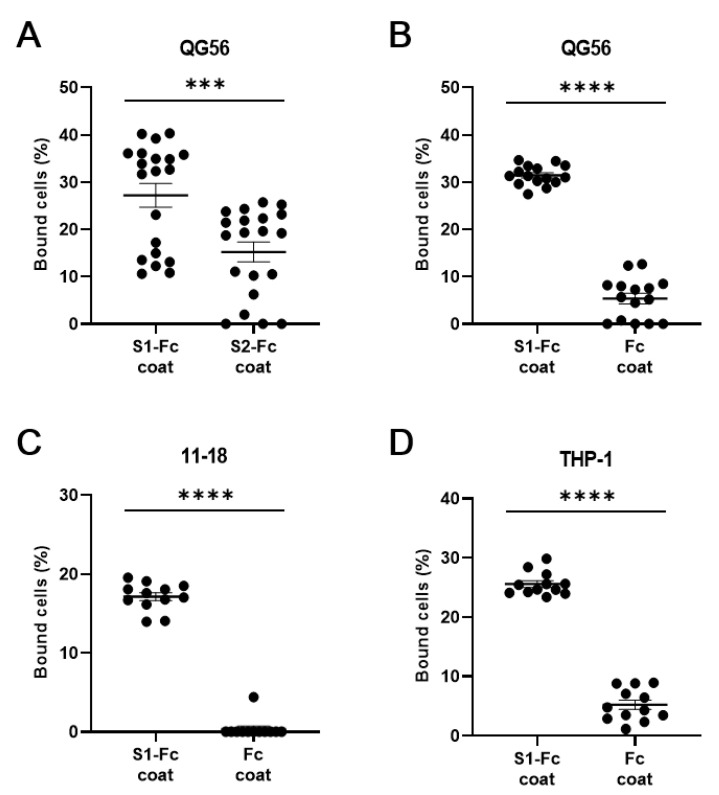
Binding of human lung epithelial cells and monocytes to S1-Fc. Cellular binding to S1-Fc was compared to S2-Fc (**A**) and to Fc alone (**B**–**D**). QG-56 (**A**,**B**), 11-18 (**C**), THP-1 (**D**) were used in this assay. Results are shown as column scatterplots overlayed with the mean ± SEM of at least 3 independent experiments, in which each sample was triplicated. *** *p* < 0.001; **** *p* < 0.0001.

As we used S1-Fc fusion protein throughout the experiments, we sought to substantiate the specific binding to the S1 protein, thereby comparatively studying the cell adhesion to S1-Fc fusion protein and two types of reference proteins S2-Fc fusion protein and IgG Fc fragment protein (Figure 4A–D). We have shown that QG-56, 11-18, and THP-1 cells hardly bound to IgG Fc fragment protein, while binding well to S1-Fc protein (Figure 4B–D). As S2-Fc protein is a scarce reagent for us, we have studied it only with QG-56 cells, which bound significantly better to S1-Fc protein than to S2-Fc protein (Figure 4A). These results confirmed the specific binding of QG-56, 11-18, and THP-1 cells to S1 protein.

**Figure 5:**
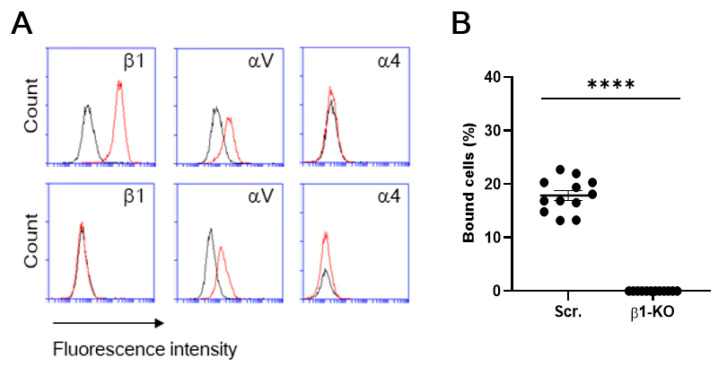
Integrin expression and S1 binding of β1-control and β1-KO cells of a human breast cancer cell line (MDA-MB-231). (**A**) The cells were stained with the fluorescently labeled mAbs to integrins. The integrin expression was analyzed by using flow cytometry for β1-control (Scr.) (upper panel) and β1-KO (lower panel) cells of MDA-MB-231. Histograms indicate the expression of integrins indicated. Data are representative of three separate experiments. Red lines, MAb; black lines, isotype. (**B**) Cellular binding to S1-Fc was compared between β1-control (Scr.) and β1-KO. Results are shown as column scatterplots overlayed with the mean ± SEM of at least 3 independent experiments, in which each sample was triplicated. **** *p* < 0.0001.

To further validate the specific interaction of β1 integrin with S1 protein, we utilized a β1 integrin knockout (KO) clone of a human breast cancer cell line (MDA-MB-231) that we previously established using CRISPR/Cas9 [16] as a model cell. A flow cytometric examination confirmed the absence of β1 integrin in the KO cells (Figure 5A). S1 binding of β1-KO cells was completely abolished, compared to that of β1-control cells (i.e., mock transfectants) [16] (Figure 5B), even though β1-KO cells retain αV integrin expression (Figure 5A). These results have revealed that β1 integrins can support binding to S1 protein independently of αV integrins.

**Figure 6:**
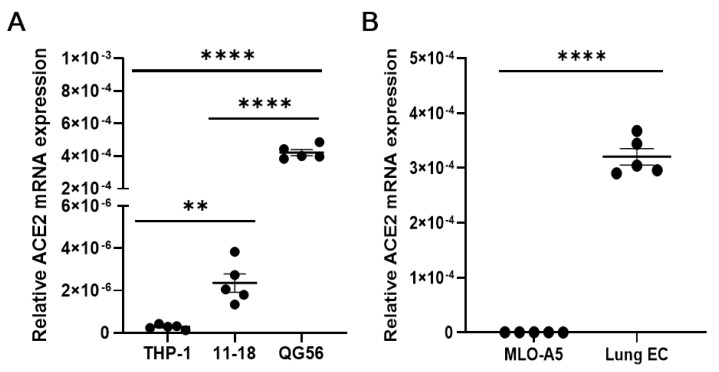
qRT-PCR measuring the expression of ACE2 in human (**A**) and mouse (**B**) cells. Relative expression of *ACE2* to *β-actin* was determined by using the 2^−ΔCT^ method. In panel A, statistical significance between two groups (THP-1 vs. 11-18) was analyzed by a two-tailed unpaired *t*-test, while other comparisons were by one-way ANOVA. Results are shown as column scatterplots overlayed with the mean ± SEM of at least 3 independent experiments, in which each sample was triplicated. ** *p* < 0.01; and **** *p* < 0.0001.

Human pulmonary epithelial cell lines and mouse lung primary cells express moderate to high levels of ACE2, the authentic receptor for the S1 protein (Figure 6A,B), which may be involved in the observed adhesive interactions of lung cells with the S1 protein. Residual binding to S1 protein in the presence of anti-integrin antibodies could be partly mediated by ACE2. As mouse ACE2 supports S1 protein binding much less efficiently than human ACE2 [21], this could be the case with human pulmonary epithelial cells. To confirm the ability of integrins (e.g., β1 integrin) to bind to the S1 protein independently of ACE2, we utilized ACE2-negative cells, such as human monocytic THP-1 cells and mouse osteoclastic MLO-A5 cells. We have shown that these ACE2-negative cells exhibited good binding to the S1 protein in a β1 integrin-dependent manner (Figure 1C and Figure 3B).

## 4. Discussion

In this study, we have demonstrated that native β1 integrins expressed on human and mouse pulmonary epithelial cells bind to the S-protein of SARS-CoV-2, thereby providing experimental evidence to support recent predictions regarding their role in this global disease [12,13]. Our results are consistent with a recent report that showed, in a cell-free ELISA-type assay, that α5β1 integrin protein immobilized on plates supported binding to the SARS-CoV-2 S-protein [15]. Our results demonstrate the critical roles played by β1 integrins in mediating cellular adhesive interactions with the S-protein, although we have shown that αV integrins on pulmonary epithelial cell lines and α4 integrins on monocytic cell lines also support binding to S1 protein. Integrins are conformationally mobile proteins that undergo dynamic structural rearrangements on the cell surface in response to intracellular signaling pathways specific to cell types [22,23]. Thus, demonstrating the ability of the S1 protein to bind integrin expressed on the surface of relevant cells is of great importance, and all of this contributes to emphasizing the significance of the results from the cell-based experiments in our study.

It has been shown in the recent report that a small molecule peptide antagonist of integrin α5β1 (ATN-161, Ac-PHSCN-NH2) inhibited the infection of SARS-CoV-2 in VeroE6 cells in vitro [15]. Due to technical difficulties, we were unable to study the ability of anti-integrin blocking antibodies to interfere with SARS-CoV-2 infection. Whereas further investigations would be necessary to confirm the ability of integrin antagonists to suppress SARS-CoV-2 infection in vitro and in vivo, integrin-targeted approaches may serve as potentially effective anti-viral treatments. A few therapeutic antibodies to integrins have been approved for the treatment of inflammatory disorders such as multiple sclerosis [24] and inflammatory bowel diseases [25]. Although proven to be clinically effective, integrin antagonism in patients can nonetheless pose serious adverse effects. The integrin inhibitor natalizumab, which blocks α4β1 and α4β7 integrins, is reported to sporadically cause progressive multifocal leukoencephalopathy, an often fatal brain disease resulting from reactivation of a latent JC virus infection due to iatrogenic immune suppression [24]. Thus, inhibition of integrins, especially β1 integrins, in clinical settings must be carried out with special precautions. Nevertheless, a multiple sclerosis patient treated with natalizumab has been reported to recover well from SARS-CoV-2 infection [26], which might be relevant to our finding that α4 integrin blockade abolished binding of S1 protein to monocytes.

Integrins have been shown to bind not only to the viral S-protein, as shown in this study, but also to the cellular ACE2, as previously reported [27]. ACE2 contains a conserved RGD sequence that is not surface exposed; however, β1 integrins bind to ACE2 in an RGD-independent manner. A recent and provocative hypothesis proposes not only that integrin binding to ACE2 sterically shields the SARS-CoV-2 binding site within ACE2, but that the cis-interaction of integrins with ACE2 on the same cells naturally prevents the virus from binding to the entry receptor ACE2. In this context, inhibition of integrins may result in the liberation of ACE2 for SARS-CoV-2 binding, potentially enhancing viral entry [14]. Although extremely intriguing, this alternative scenario appears to be unlikely [6]. Simultaneous inhibition of integrin–S-protein interactions and the ACE2–S-protein interactions offers a promising therapeutic approach for robustly suppressing SARS-CoV-2 infections [6]. We have shown that β1 integrin supports the binding of S1 protein to mouse primary lung cells.

## 5. Conclusions

To conclude, we have provided important experimental evidence that SARS-CoV-2 spike protein 1 binds to β1 integrins on the surface of pulmonary epithelial cells. β1 integrins bind to the S1 protein independently of ACE2; however, the therapeutic potential of integrin antagonists inhibiting SARS-CoV-2 infections with or without an intervention targeting ACE2 in vivo warrants further investigation.

## Data Availability

Not applicable.

## Abbreviations

SARS-CoV-2	severe acute respiratory syndrome coronavirus 2
ACE2	angiotensin-converting enzyme 2
RGD	arginyl–glycyl–aspartic acid
Q-RT-PCR	quantitative reverse transcription polymerase chain reaction
ELISA	enzyme-linked immunosorbent assay

## References

[1] Lai C.C., Shih T.P., Ko W.C., Tang H.J., Hsueh P.R. (2020). Severe acute respiratory syndrome coronavirus 2 (SARS-CoV-2) and coronavirus disease-2019 (COVID-19): The epidemic and the challenges. Int. J. Antimicrob. Agents.

[2] Phua J., Weng L., Ling L., Egi M., Lim C.-M., Divatia J.V., Shrestha B.R., Arabi Y.M., Ng J., Gomersall C.D. (2020). Intensive care management of coronavirus disease 2019 (COVID-19): Challenges and recommendations. Lancet Respir. Med..

[3] Johns Hopkins University Coronavirus Resource Center COVID-19 Dashboard by the Center for Systems Science and Engineering (CSSE) at Johns Hopkins University (JHU). https://coronavirus.jhu.edu/map.html.

[4] Yan R., Zhang Y., Li Y., Xia L., Guo Y., Zhou Q. (2020). Structural basis for the recognition of SARS-CoV-2 by full-length human ACE2. Science.

[5] Monteil V., Kwon H., Prado P., Hagelkrüys A., Wimmer R.A., Stahl M., Leopoldi A., Garreta E., Del Pozo C.H., Prosper F. (2020). Inhibition of SARS-CoV-2 Infections in Engineered Human Tissues Using Clinical-Grade Soluble Human ACE2. Cell.

[6] Yan S., Sun H., Bu X., Wan G. (2020). New Strategy for COVID-19: An Evolutionary Role for RGD Motif in SARS-CoV-2 and Potential Inhibitors for Virus Infection. Front. Pharmacol..

[7] McKee D.L., Sternberg A., Stange U., Laufer S., Naujokat C. (2020). Candidate drugs against SARS-CoV-2 and COVID-19. Pharmacol. Res..

[8] Pirone L., Del Gatto A., Di Gaetano S., Saviano M., Capasso D., Zaccaro L., Pedone E. (2020). A Multi-Targeting Approach to Fight SARS-CoV-2 Attachment. Front. Mol. Biosci..

[9] Clausen T.M., Sandoval D.R., Spliid C.B., Pihl J., Perrett H.R., Painter C.D., Narayanan A., Majowicz S.A., Kwong E.M., McVicar R.N. (2020). SARS-CoV-2 Infection Depends on Cellular Heparan Sulfate and ACE2. Cell.

[10] Park E.J., Yuki Y., Kiyono H., Shimaoka M. (2015). Structural basis of blocking integrin activation and deactivation for anti-inflammation. J. Biomed. Sci..

[11] Shimaoka M., Takagi J., Springer T.A. (2002). Conformational Regulation of Integrin Structure and Function. Annu. Rev. Biophys. Biomol. Struct..

[12] Sigrist C.J., Bridge A., Le Mercier P. (2020). A potential role for integrins in host cell entry by SARS-CoV-2. Antivir. Res..

[13] Tresoldi I., Sangiuolo C.F., Manzari V., Modesti A. (2020). SARS-COV-2 and infectivity: Possible increase in infectivity associated to integrin motif expression. J. Med. Virol..

[14] Luan J., Lu Y., Gao S., Zhang L. (2020). A potential inhibitory role for integrin in the receptor targeting of SARS-CoV-2. J. Infect..

[15] Beddingfield B.J., Iwanaga N., Chapagain P.P., Zheng W., Roy C.J., Hu T.Y., Kolls J.K., Bix G.J. (2021). The Integrin Binding Peptide, ATN-161, as a Novel Therapy for SARS-CoV-2 Infection. JACC Basic Transl. Sci..

[16] Kawamoto E., Nago N., Okamoto T., Gaowa A., Masui-Ito A., Akama Y., Darkwah S., Appiah M.G., Myint P.K., Obeng G. (2021). The Lectin-Like Domain of Thrombomodulin Inhibits β1 Integrin-Dependent Binding of Human Breast Cancer-Derived Cell Lines to Fibronectin. Biomedicines.

[17] Skillrud D.M., Martin W.J. (1984). The isolation of rat alveolar type II cells: A simplified approach using Percoll density centrifugation. Lung.

[18] Hansen T., Chougule A., Borlak J. (2014). Isolation and cultivation of metabolically competent alveolar epithelial cells from A/J mice. Toxicol. Vitr..

[19] Weetall M., Hugo R., Maida S., West S., Wattanasin S., Bouhel R., Weitz-Schmidt G., Lake P., Friedman C. (2001). A Homogeneous Fluorometric Assay for Measuring Cell Adhesion to Immobilized Ligand Using V-Well Microtiter Plates. Anal. Biochem..

[20] Sheppard D. (2003). Functions of Pulmonary Epithelial Integrins: From Development to Disease. Physiol. Rev..

[21] Dinnon K.H., Leist S.R., Schäfer A., Edwards C.E., Martinez D.R., Montgomery S.A., West A., Yount B.L., Hou Y.J., Adams L.E. (2020). A mouse-adapted model of SARS-CoV-2 to test COVID-19 countermeasures. Nature.

[22] Li J., Su Y., Xia W., Qin Y., Humphries M.J., Vestweber D., Cabañas C., Lu C., Springer T.A. (2017). Conformational equilibria and intrinsic affinities define integrin activation. EMBO J..

[23] Li J., Springer T.A. (2017). Energy landscape differences among integrins establish the framework for understanding activation. J. Cell Biol..

[24] Kawamoto E., Nakahashi S., Okamoto T., Imai H., Shimaoka M. (2012). Anti-Integrin Therapy for Multiple Sclerosis. Autoimmune Dis..

[25] Danese S., Vuitton L., Peyrin-Biroulet L. (2015). Biologic agents for IBD: Practical insights. Nat. Rev. Gastroenterol. Hepatol..

[26] Aguirre C., Meca-Lallana V., Barrios-Blandino A., del Río B., Vivancos J. (2020). Covid-19 in a patient with multiple sclerosis treated with natalizumab: May the blockade of integrins have a protective role?. Mult. Scler. Relat. Disord..

[27] Clarke N.E., Fisher M.J., Porter K.E., Lambert D.W., Turner A.J. (2012). Angiotensin Converting Enzyme (ACE) and ACE2 Bind Integrins and ACE2 Regulates Integrin Signalling. PLoS ONE.

